# A protease-sensing circuit links neutrophil inflammation to virulence regulation in *Streptococcus pyogenes*

**DOI:** 10.64898/2026.05.15.725401

**Published:** 2026-05-15

**Authors:** Stephanie Guerra, Ananya Dash, Doris L. LaRock, Christopher N. LaRock

**Affiliations:** 1 Department of Microbiology and Immunology, Emory University School of Medicine, Atlanta, GA; 2 Microbiology and Molecular Genetics Program, Laney Graduate School, Emory University, Atlanta, GA; 3 Immunology and Molecular Pathogenesis Program, Laney Graduate School, Emory University, Atlanta, GA; 4 Division of Infectious Diseases, Department of Medicine, Emory University School of Medicine, Atlanta, GA

**Keywords:** *Streptococcus pyogenes*, invasive infection, innate immunity, neutrophils, neutrophil extracellular traps, virulence factor, protease, gene regulation

## Abstract

*Streptococcus pyogenes* (Group A *Streptococcus*) causes infections with a disproportionately hyperinflammatory response from the host, such as scarlet fever, necrotizing fasciitis, and toxic shock syndrome. Inflammation is specifically driven by *S. pyogenes* virulence factors, including the protease SpeB, but how inflammation impacts SpeB expression in return during disease is unknown. In this study, we identify a novel interaction between NETosis, a form of inflammatory cell death for neutrophils, and the induction of *speB*. Specifically, while the cathelicidin peptide LL-37 can repress *speB* through the two-component regulatory system CovRS, neutrophil proteases released during NETosis relieve repression of *speB* by degrading another repressor of *speB*, the bacterial protein Vfr. Furthermore, at high cell densities, SpeB autoregulates its expression through similar degradation of Vfr. Abrogating the formation of NETs or depleting neutrophils resulted in *speB* repression *in vivo*, showing the mutual host and pathogen counterattacks collectively lead to the pathological exacerbations characteristic of disease.

## Introduction

*Streptococcus pyogenes* (*Spy*; Group A Streptococcus) is a human-exclusive bacterial pathogen responsible for billions of infections and more than 500,000 deaths annually, making it one of the top ten infectious causes of human mortality worldwide^1^. Most infections are relatively mild, such as impetigo and pharyngitis, but serious, and often fatal diseases like bacteremia, puerperal sepsis, Streptococcal toxic shock syndrome (STSS), and necrotizing fasciitis arise when *Spy* becomes invasive. Virulence factors play a major role in disease progressing by facilitating tissue invasion, evading immune cell killing, and creating intracellular reservoirs^6^.

SpeB is a major virulence factor conserved amongst *Spy*, and is essential for colonization and proliferation within tissue in a variety of infection models^7^. Nonetheless, mutants arise during severe infections that, enigmatically, no longer express *speB*^8,9^. These mutations are most commonly in the two-component system CovRS (CsrRS), which modulates expression of nearly 15% of *Spy* genes, including most virulence factors^10–12^. CovRS senses the host cathelicidin peptide LL-37, produced by epithelial and immune cells as part of innate immune defense and wound repair^5,13–15^. Loss-of-function *covRS* mutations dysregulate their virulence factors in a manner similar to constitutive LL-37 induction, decreasing production of SpeB, while increasing production of capsule (through the *hasABC* operon) and toxins like SLO, NADase, Mac/IdeS, and ^4^. The major inducer of *speB* is RopB in response to the quorum-sensing peptide SIP^16,17^. *In vitro*, the virulence factor-related (Vfr) protein also appears to antagonize induction, on the basis that *vfr* mutants express greater *speB*^18,19^. How the combination of these possible host and microbial signals are integrated to regulate SpeB during infection is unknown.

This work shows that *Spy* establishes a phenotypically diverse population during infection through the heterogeneous expression of *speB*. Using genetics, RopB, Vfr, and the CovRS system are all found to be essential for generating discrete SpeB-producing and non-producing subpopulations. We show that Vfr is a labile sensor of protease activity that is degraded by SpeB, allowing it to autoregulate it’s own expression by relieving Vfr repression. Furthermore, neutrophil serine proteases also relieve Vfr repression, allowing the bacteria to sense both influx of neutrophils and their activities, with maximal *speB* induction occurring in response to neutrophil extracellular traps (NETs). Despite neutrophils being the major producers of LL-37, which can lead to *speB* repression through CovRS, protease sensing by Vfr ensures SpeB, which is important for resisting neutrophil-produced antimicrobials including LL-37^20^, is still expressed in its presence. Together, our results show how *Spy* navigates the challenge of expressing the right virulence factor at the right time by use of a circuit that integrates host and microbial cues.

## Results

### *S. pyogenes* establishes subpopulation heterogeneity in their expression of *speB*.

**Fig. 1.**
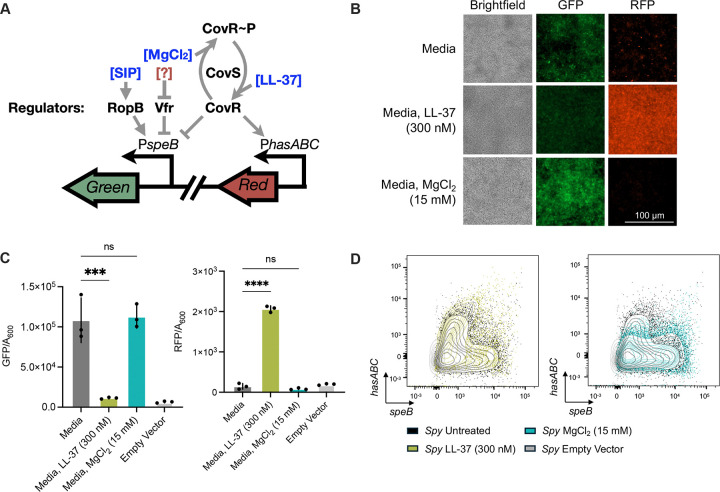
*S. pyogenes* establishes subpopulation heterogeneity in their expression of *speB*. (A) *speB* regulatory circuit schematic. RopB induces *speB* expression in the presence of SIP peptide and Vfr inhibits *speB* expression by blocking RopB-SIP complex. CovS kinase-mediated CovR phosphorylation (CovR~P) derepresses *speB*, while CovS phosphatase-mediated CovR dephosphorylation represses *speB*. MgCl_2_ and LL-37 mediate CovS kinase and phosphatase, respectively. Induction of *speB* expression was evaluated with GFP fluorescence and *hasABC* expression was evaluated with RFP fluorescence. (B, C, D) Wild-type *Spy* was treated with LL-37 (300 nM) or MgCl_2_ (15 mM). (B) Live-cell fluorescent microscopy with brightfield, GFP, and RFP channels on *Spy* culture grown at stationary phase. Scale bars, 100 μm. (C) Measurement of fluorescence over cell density after 10 h of growth. (D) Flow cytometry demonstrating *speB* expression (GFP; horizontal axis) and *hasABC* expression (RFP; vertical axis) of *Spy* growing at stationary phase. Statistical significance was determined using a one-way ANOVA with Dunnett’s multiple comparisons test. ****P<0.0001, ***P<0.001, ns: not significant.

To examine *speB* expression within *Spy*, we generated a transcriptional fusion of *gfp* with 1000 bp upstream of the *speB* to include P1 and P2 regions of the *speB* promoter^16^. The best-characterized regulator of *speB* is CovRS; *speB* is derepressed when CovR is phosphorylated (CovR~P), in contrast to the capsule biosynthesis operon *hasABC*, which is induced when CovR is unphosphorylated^12^. CovR phosphorylation depends on the histidine kinase, CovS, which is sensitive to environmental signals. To examine the contribution of *speB* inducers separate from this repressor, we created a tandem reporter that also has the *hasABC* promoter fused to *rfp*^21^ (Fig. 1A). To test the accuracy and precision of the reporter construct, *Spy* was grown in concentrations of LL-37 (300 nM) or MgCl2 (15 mM) that did not impact bacterial growth (**Fig. S1A**), but which divergently regulate CovR phosphorylation of CovS^12^. As expected for a quorum-sensing regulated protease^17^, GFP (*speB*) is induced as *Spy* approaches late log phase. The addition of LL-37 induced RFP (*hasABC*) and repressed both the expression of GFP (*speB*) (Fig. 1C) as well as production of the mature, active protease (**Fig. S1B**). Contrarily, GFP (*speB*) induction was maintained in the presence of MgCl2, but RFP (*hasABC*) repressed. Examination by fluorescence microscopy recapitulated these observations, but suggested heterogeneity in these responses within the bacterial population (Fig. 1B).

To quantify this at the single-cell level, we next used flow cytometry. Size, BactoView^™^ Dead 760/780, and an antibody against the *Spy*-specific antigen Group A Carbohydrate (anti-GAC), were used to gate on single, live cells (**Fig. S1C**). As expected, *Spy* grown with LL-37 had a population shift positive for RFP and negative for GFP (Fig. 1D). Conversely, growth with MgCl_2_ resulted in a lower and rightward shift in the population. However, there was significant heterogeneity even in these induced conditions, with large populations of intermediate expression.

**Fig. 2.**
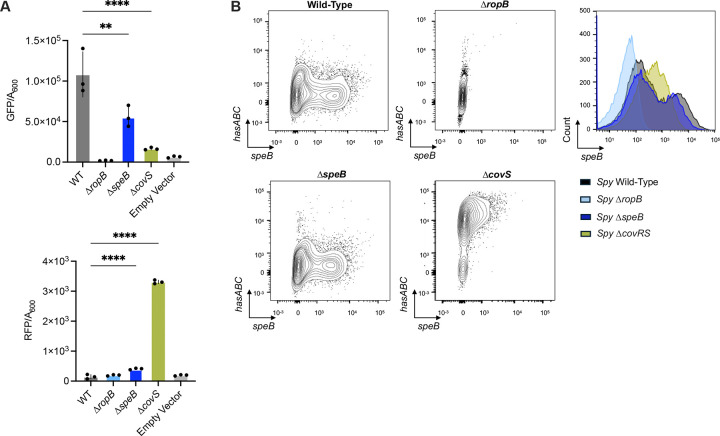
*S. pyogenes* regulators are required for heterogenous *speB* expression. *Spy* genetic control strains of the *speB* regulatory circuit were evaluated. (A) Measurement of fluorescence over cell density after 10 h of growth. (B) Flow cytometry demonstrating *speB* expression (GFP; horizontal axis) and *hasABC* expression (RFP; vertical axis) of *Spy* growing at stationary phase. Statistical significance was determined using a one-way ANOVA with Dunnett’s multiple comparisons test. ****P<0.0001, **P<0.01, ns: not significant.

Genetic knockouts of known and probable SpeB regulators (Fig. 1A) were created to further validate the fluorescent reporters while defining determinants of heterogenicity. None impacted bacterial growth (**Fig. S2A**). A *ΔcovS* mutant constitutively expressed high *hasABC*, (RFP), while repressing *speB*, as expected. A *ΔropB* mutant did not express *speB*, in agreement with its reported importance for *speB* induction^17^. Interestingly, a *ΔspeB* mutant also showed less induction of the *speB* reporter, suggesting the possibility of some autoregulation (Fig. 2A). Reporter fluorescence examined by cytometry (Fig. 2B) and microscopy (**Fig. S2B**) were consistent with these observations. Similarly, the *ΔspeB* and Δ*covS* mutants expressed slightly less, while *ΔropB* bacteria expressed little (Fig. 2B, **S2B**).

### Vfr is a dominant repressor for *speB*.

**Fig. 3.**
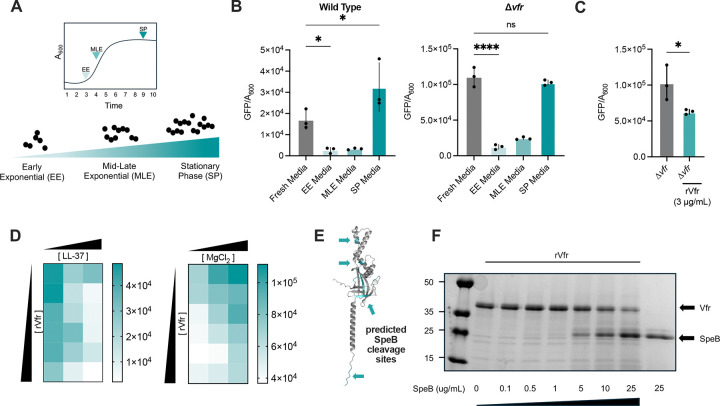
Vfr is a dominant repressor for *speB*. (A, B) Wild-type *Spy* was grown to early exponential (EE), mid-late exponential (MLE), and stationary phase (SP). Spent media from each stage of growth were collected and filter sterilized through 0.2μm filter and inoculated with *Spy* to detect GFP (*speB*) and RFP (*hasABC*) fluorescence. (C) *Spy Δvfr* was grown in the presence or absence of recombinant Vfr (rVfr). (D) Wild-type *Spy* was grown with rVfr (0 – 10 ug/mL) and either LL-37 (0 – 300 nM) or MgCl2 (0 – 15 mM) for 10 h. (E) AlphaFold structure of Vfr with potential SpeB cleavage sites (blue). (F) SDS-PAGE of Vfr (0.3 mg/mL) incubated with incremental concentrations of SpeB for 2.5 h. Statistical significance was determined using a one-way ANOVA with Dunnett’s multiple comparisons test. ****P<0.0001, *P<0.05, ns: not significant.

Since secreted bacterial factors, including the SIP quorum-sensing peptide, give the potential for bacteria to coordinate expression within the population, we next performed media swap experiments. Wild-type *Spy* was grown to early exponential (EE), mid-late exponential (MLE), or stationary phase (SP) and the spent media removed, filtered, then used to supplement wild-type *Spy* growth carrying the SpeB reporter (Fig. 3A). As expected, since the SIP inducer accumulates in stationary phase cultures where SpeB is optimally expressed, SP spent media induced GFP (*speB*) (Fig. 3B). Surprisingly, however, growth from earlier growth phase cultures not only lacked *speB* inducer activity but suppressed its expression (Fig. 3B). This suggested that these cells produced a soluble factor that dominantly repressed the ability of other cells to produce SpeB. We next repeated the media swap experiment using a *Δvfr* mutant, which highly expressed SpeB (**Fig. S2B**), to see if a soluble factor from wild-type *Spy* could repress this. Spent media from only early-growth phase cultures could significantly repress this (Fig. 2B).

To assess whether it was Vfr itself in the media suppressing *speB*, we examined the activity of recombinant Vfr (rVfr). rVfr reduced GFP (*speB*) expression significantly in *SpyΔvfr* (Fig. 3C). To assess the relative phenotypic dominance of SpeB regulation by autoregulatory mechanisms (Rop-SIP-Vfr dependent) versus environmental signaling mechanisms (CovRS-dependent), wild-type *Spy* containing the dual reporter was grown in the presence of CovRS inducers (LL-37 or MgCl_2_) and Vfr at increasing concentrations. Both LL-37 and Vfr function synergistically in repressing *speB*, while Vfr antagonized MgCl_2_-mediated induction of *speB* (Fig. 3D). Notably, *speB* expression in *Spy Δvfr* is unaffected by LL-37 or MgCl_2_, further validating its dominance over CovRS regulation (**Fig. S2C**).

Interestingly, the Vfr structure contains several potential protease SpeB cleavage sites (Fig. 3E)^22^. Since degradation could explain the SpeB-dependent changes in GFP (*speB*) expression, rVfr was incubated in the presence and absence of purified SpeB. Vfr degradation was observed in a concentration-dependent manner (Fig. 3F). Together, these data highlight that Vfr is a SpeB-labile repressor of SpeB expression.

### *speB* is induced in the presence of immune effectors.

**Fig. 4.**
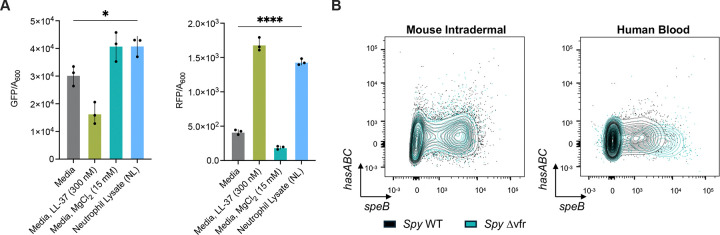
*speB* expression is induced in the presence of immune effectors. (A) Evaluating the effects of neutrophil lysate on *speB* and *hasABC* expression. *Spy* grew in the presence of neutrophil lysates (10^6^ cells/mL) compared to *Spy* growing in RPMI 5% THY. Treatment with LL-37 (300 nM) and MgCl_2_ (15 mM) served as controls. (B) Flow cytometry demonstrating *speB* (GFP fluorescence; horizontal axis) and *hasABC* induction (RFP fluorescence; vertical axis) of 10^8^ CFU of *Spy* during mouse intradermal and human blood infections after 24 h and 4 h, respectively.

Neutrophils are the first line of defense during innate immune response and are quickly recruited during bacterial infections. Neutrophils highly express the immune effector LL-37^5^, which *Spy* recognizes through CovRS, which by current models should repress *speB* expression. *Spy* grown with neutrophil lysates induced *hasABC* expression, consistent with CovRS stimulation, but did not repress *speB* expression (Fig. 4A). Similarly, in the mouse invasive infection model and in whole human blood, *Spy* induced *hasABC* while populations of high *speB* expressors were maintained (Fig. 4B). *Spy* with regulator knockouts were also evaluated to determine consistency in mouse and blood tissue infections with *in vitro* regulation. *Δvfr* maintained high *speB* expression, *ΔropB* bacteria were negative for *speB* expression, and *ΔcovS* contained a *hasABC* high population (**Fig. S3A**).

### Immune effectors induce *speB* through Vfr.

**Fig. 5.**
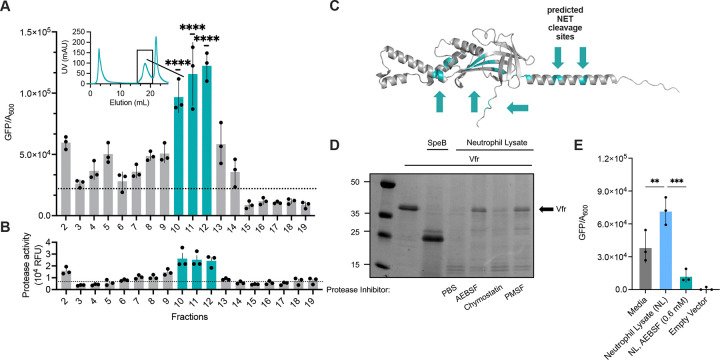
Immune effectors induce *speB* through Vfr. (A, B) Neutrophil lysates were fractioned based on net surface charge through anionic exchange. (A) Fractions were used to supplement *Spy* growth and *speB* expression was evaluated with GFP fluorescence at late log phase. Protein content within each elution (mL) was detected by UV (mAU) (Upper left). (B) Fractions were also used to evaluate protease activity. (C) AlphaFold structure of Vfr with potential NET protease cleavage sites (blue). (D) SDS-PAGE of Vfr (0.3 mg/mL) incubated with lysate from neutrophils (10^6^ cells/mL) treated with inhibitors AEBSF, Chymostatin, or PMSF. PBS served as a vehicle control. (E) *Spy* grew in the presence of neutrophil lysates (10^6^ cells/mL) and AEBSF (0.6 mM) compared to *Spy* growing in RPMI 5% THY. Statistical significance was determined using a one-way ANOVA with Dunnett’s multiple comparisons test. ****P<0.0001, ***P<0.001, **P<0.01.

This high-level of expression *in vivo* suggested that *speB* could be induced, not just repressed, in the presence of immune effectors. Along with LL-37, neutrophil granules store many major inflammatory components secreted during infections^23,24^. To assess whether other neutrophil derived molecules play a role in *speB* regulation, neutrophil lysates were fractionated and examined for their ability to induce or repress *speB* expression. Upon supplementation, GFP (*speB)* was induced by three fractions (10–12) containing active proteases (Fig. 5AB).

While *Spy* is resistant NET killing^25,26^, the immune effectors released by neutrophils during NETosis, include not only LL-37, but proteases like neutrophil elastase (NE)^27^. Based on the protease profile of NETs^28^, Vfr contains several potential sites for cleavage (Fig. 5C). Neutrophils degraded rVfr (Fig. 5D), as did purified NE (**Fig. S3C**). The NE-specific inhibitor, BAY-678, was not sufficient to inhibit neutrophil lysate-mediated rVfr degradation, but serine inhibitors AEBSF and PMSF did (Fig. 5D, **S3C**), suggesting a sufficiency but not requirement for other neutrophil proteases. Moreover, *Spy* grown with neutrophil lysates and inhibitor AEBSF had resulted in *speB* inhibition expression, compared to neutrophil lysate alone (Fig. 5E). Notably, these conditions maintained *hasABC* expression, consistent with CovRS stimulation (**Fig. S3D**). Altogether, these data suggest Vfr is a substrate for both bacterial and host-mediated degradation.

### SpeB and neutrophils are sufficient to induce *speB* expression.

**Fig. 6.**
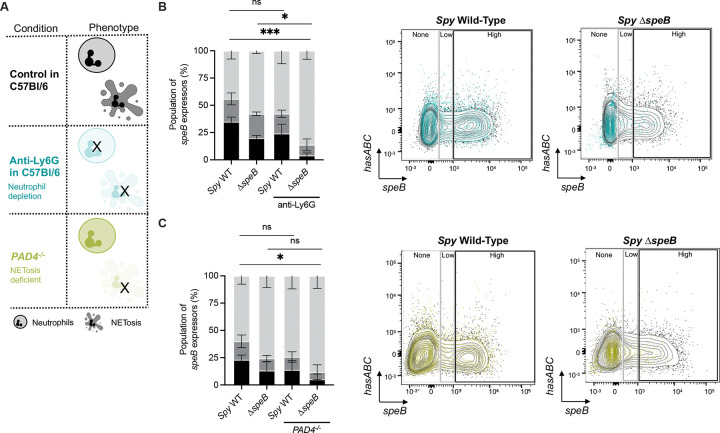
SpeB and neutrophils are sufficient to induce *speB* expression. (A) Diagram of mouse intradermal infection model in three different conditions: neutrophil-depleted mouse (anti-Ly6G treatment); NET-deficient mouse (*PAD4*^−/−^); and neutrophil competent (wild-type) control. (B, C) Flow cytometry on mouse skin lesions from 24 h intradermal infection of 10^8^ CFU of *Spy* or *Spy ΔspeB*. Population of *speB* expressors was determined based on fluorescent intensity during flow cytometry. No expressors range was based on the negative empty vector control. (B) C57Bl/6 mice were treated with neutrophil-depleting antibody, anti-Ly6G, (blue) or control (black) for 24 h then infected. (C) NETosis-deficient mice (*PAD4*^−/−^, yellow) or control (C57Bl/6 mice, black) were infected. Statistical significance of high *speB* expressors was determined using a two-way ANOVA with Dunnett’s multiple comparisons test. ***P<0.001, *P<0.05, ns: not significant.

To examine the impact that neutrophils have on *speB* regulation *in vivo*, neutrophils were depleted by anti-Ly6G treatment, as previously^29^, significantly decreasing their numbers (**Fig. S4A**). After a 24 h intradermal infection, *Spy* from the tissue was examined by flow cytometry for *speB* expression, relative to a *Δvfr* control to define the high-expressing population (**Fig. S4B**). *Spy* expression of *speB* was only moderately decreased in the absence of neutrophils (Fig. 6B). However, infection of neutropenic mice with a *ΔspeB*, where there is neither host or microbial proteases to cleave Vfr, prevented *speB* expression (Fig. 6B). Mice deficient in peptidyl-arginine deiminase 4 (PAD4) are unable to form NETs^30^. To separately examine NET contributions *PAD4*^−/−^ mice were infected with *Spy* and *speB* expression examined. SpeB expression was reduced in *PAD4*^−/−^ mice, additively with a contribution of SpeB itself (Fig. 6C). Collectively, this data suggests that *Spy* integrates the sensing of both neutrophils and feedback from SpeB to titrate expression during infection.

## Discussion

Since a bacterium must adapt to environmental changes, they modulate expression in response to diverse stimuli, including stress, metabolites, quorum sensing, pH, and temperature. The niche of *Spy* is exclusively the human, potentially limiting its exposure to the range that some other species face. Yet within this host, it is nonetheless presented with challenges. Foremost amongst these are the immune defenses, which escalate through the course of infection. Fine-tuning its regulation toward the virulence factors needed to overcome this specific challenge is key to the species. SpeB is a potent pro-inflammatory virulence factor required to establish infection and has key roles in determining the course and severity of infections^6^. Accordingly, the regulation of SpeB must be tied to circumstances where it will be advantageous. Regulation of *speB* is thus multifactorial, as it integrates sensors to promote colonization and survival. In this study, we highlight a novel mechanism for SpeB regulation through Vfr detection of neutrophils and their activation state. Our data shows that neutrophils, despite functioning as an LL-37 reservoir expected to repress *speB* expression through the CovRS system, actually induces it during invasive skin infections.

The relative contributions of these regulators could vary by disease^31^. In response to inflammation, neutrophils are recruited to infection sites to engulf and inactivate pathogens. Despite neutrophils playing a major role in combating most infections, several studies indicate they can worsen *Spy* infections, in particular, in the upper respiratory tract^29,32^. Here, RopB can detect the SIP homologs used for quorum sensing by other *Streptococci* species of the upper respiratory microbiota^33^. This ensures SpeB expression at low cell densities and could allow SpeB expression even before neutrophil infiltration. Importantly, the type of CovS mutants that interfere with SpeB expression do not occur during human pharyngitis or experimental mouse models of it, and *ΔspeB* mutants are highly attenuated, indicating its essentiality at this site^29^. This would subsequently require SpeB expression during infections, suggesting repression by LL-37, released by the abundant neutrophils recruited during infection, is, at best, limited.

Our work suggests that Vfr functions as a biosensor of protease activity that regulates the major protease of *Spy*, SpeB. It is additionally degraded by neutrophil proteases, thereby relieving repression in the presence of these important immune cells. Originally identified through a transposon mutagenesis screen^18^, Vfr has been suspected to regulate SpeB in a RopB-dependent manner^19^. Our results showing that Vfr functions as a negative regulator of *speB* early during growth in culture is consistent with this, but the mechanism in which SIP quorum sensing relieves Vfr-mediated repression at late and stationary growth remains to be elucidated. Since the SpeB protease itself can degrade Vfr, autoregulation may play a role in this temporal regulation. However, it is not clear whether this would be relevant during infection, since the host can provide the proteases to overcome this. Similarly, CovRS mediates repression of SpeB in some *in vitro* conditions, such as with LL-37 alone. However, since repression is not maintained in the presence of the major LL-37-producing cells, neutrophils, the effect may be more limited during infections, either due to heterogeneity in the population or dominant effects of Vfr. Importantly, other than releasing LL-37, neutrophils undergoing NETosis or degranulating release proteases, including NE^34^, that degrade Vfr. Together, this highlights the importance of *in vivo* data for examining pathways such as the regulation of SpeB, since pathways sensing multiple bacterial and host factors can intersect.

In summary, we demonstrate how *Spy* overcomes challenges in the temporal regulation of the virulence factor SpeB by a circuit that integrates multiple considerations in the host-pathogen interactions. Clear Vfr homologues can be found throughout the Streptococci (*S. agalactiae, S. urinalis, S. pseudoporcinus, S. porcinus, S. uberis, S. iniae, S. equi, S. dysgalactiae, S. canis*), despite none of these species encoding a SpeB homolog. This suggests that Vfr may be a regulator module more generally, and that through these other species senses broader factors as well. We propose a model wherein *Spy*, through the Vfr protein, coordinates expression of SpeB within the bacterial population. At high cell densities, quorum sensing is sufficient to induce expression. However, Vfr allows expression at lower cell densities if there is sufficient protease to degrade it. This can be accomplished if there are some SpeB-expressing cells already, thus leading to further coordination between cells independent of classic quorum-sensing, or, an override in the instance of proximate protease-producing immune cells. This further allows heterogeneity within the population, as each cell is exposed to different concentrations of SIP, LL-37, VFR, and protease. Altogether, beyond demonstrating a previously undescribed mechanism for regulating a protease through a natural biosensor for its activity, we show that this integrated regulation poises the pathogen to respond to shifts in innate immune pressure as part of the sophisticated virulence strategy of *Spy*.

### Resource Availability

#### Lead contact.

Requests for further information and resources should be directed to and will be fulfilled by the lead contact, Christopher LaRock (christopher.larock@emory.edu)

#### Materials availability.

All unique reagents generated in this study are available from the lead contact upon completion of a material transfer agreement.

#### Data and code availability.

This study did not generate any original sequence data or code. Any additional information required to reanalyze the data reported in this study is available from the lead contact upon request.

## Methods

### Bacterial strains and growth conditions:

All strains are described in Table 1. *Spy* strains were routinely grown in Todd Hewitt broth with 5% yeast (THY) at 37°C with 5% CO_2_. Bacterial aliquots were washed in PBS and resuspended in PBS with 20% glycerol for storage at −80°C and grown fresh for each experiment. The bacterial mutations *ΔcovS, ΔropB, and Δvfr* were obtained through lambda red recombineering, as described previously^39^. Briefly, a kanamycin resistance cassette was PCR amplified with the primer sequences outlined in Table 2, each carrying 5’ homology to the chromosomal sequence flanking the sites of the desired mutation. *Spy* 5448 carrying recombineering plasmid pAV488 were electroporated with the PCR product and selected for kanamycin resistance. Curing of the recombineering plasmid was achieved by selecting colonies susceptible to chloramphenicol. Gene knockout and lack of spurious secondary-site mutations were validated by whole-genome sequencing (Plasmidsaurus). Reference sequences and plasmids are detailed in Table 3.

### Plasmids:

Fluorescent reporter plasmids pDCerm-*PspeB::GFP*, pDCerm*-PhasA::RFP*, and pDCerm-*PspeB::GFP-PhasA::RFP* were created for this study by Polymerase Incomplete Primer Extension (PIPE) cloning technique, as previously described^40^. *Spy* promoters were amplified from 5448, GFP from (GenBank: OM212390.1), and RFP from (GenBank: KM521211.1) for insertion into pDCerm^41^ using the primers in Table 2. The sequence was validated using whole plasmid sequencing (Plasmidsaurus) and constructs transformed into each *Spy* strain by electroporation. Expression vector pETxSUMO-Vfr was created using primers Vfr F and Vfr R to amplify *vfr* from GAS 5448 and the previously described PIPE primers pETxSUMO F and pETxSUMO R to amplify the expression vector^40^. Reference sequences and plasmids are detailed in Table 3.

### Fluorescence during growth:

*Spy* 5448 strains grown in Todd-Hewitt Broth with 5% yeast (THY) to mid-exponential phase were used to inoculate fresh, phenol red-free RPMI supplemented with THY (5%) and erythromycin (2 μg/mL) in a 96-well black, clear-bottom plate (Costar). Cultures were grown for 10 hours at 37 °C and 5% CO_2_ with measurements of absorbance (600 nm), GFP (ex. 479 nm, em. 520 nm), and RFP (ex. 579 nm, em. 616 nm) using a BioTek Synergy H1 plate reader. Expression of *speB* and *hasABC* were analyzed by fluorescence of GFP or RFP over absorbance. Supplementation with LL-37 300 nM (GeneScript) and MgCl_2_ 15 mM (Sigma M9272).

### Whole blood and neutrophil experiments:

Whole blood was collected from healthy adult donors with informed consent and approval from the Emory University’s Children’s Clinical and Translational Discovery Core. For infections, 10^8^ CFU bacteria suspended in 100 μL of PBS and used to inoculate 400 μl of whole blood. Inoculated blood was incubated on a rotisserie mixer for 4 h. After 4 h, to lyse host cells, the inoculum was treated with Triton X 0.05% (Sigma T8787) for 15 minutes, then samples were stained and fixed for analysis. For neutrophil experiments, neutrophils were isolated from whole human blood by centrifugation in PolymorphPrep (AxisShield) as previously^42^, then diluted in RPMI. To obtain protein content from neutrophils, suspension with neutrophils in RPMI were lysed via sonication (11% amplitude for 4 minutes at 30 second intervals) and centrifuged at 6,000 × g for 5 minutes to remove cellular debris. Neutrophil lysates were used for plate reader analysis or SDS PAGE. Serine protease inhibitor AEBSF 0.6 mM (Sigma 508436) was used for plate reader analysis.

### Animal experiments:

All animal use and procedures were performed with approval from Emory Institutional Animal Care and Use Committee. Mice were housed in specific pathogen-free conditions with a 14 h light/10 h dark cycle in a standard ambient environment (~20 °C and ~50% humidity) in ABSL-2 conditions. Experiments were performed using both male and female wild-type C57Bl/6 and NET-deficient *PAD4*^−/−^ (JAX #030315)^30^ mice of 8–12 weeks of age (Jackson Laboratories). There was no attrition or drop out of subjects. Animal were assigned to experimental groups using simple randomization. In experiments where neutrophils were depleted, 50 μg anti-Ly6G (1A8) (BioXCell) or PBS vehicle control were delivered intraperitoneally 24 h before infection. Depletion was confirmed by flow cytometry using Ly6G (Invitrogen 367-9668-82), CD11b (Invitrogen 63-0112-82), and CXCR1 (RD Systems FAB8628P) antibodies as previously^29^. *Spy* 10^8^ CFU were suspended in 100 μL of PBS and injected subcutaneously, as previously^38^. After 24 h, mice were euthanized, lesions excised and mechanically homogenized, then stained and fixed for analysis.

### Flow Cytometry:

All samples were pelleted at 12,000 × g and washed with PBS with 1 mM EDTA. For viability, samples were stained using BactoView^™^ Dead 760/780 (Biotium cat. 40113) as per manufacturer protocol, then strained through a 100 μm filter (Avantor). Samples were fixed using 4% paraformaldehyde for 30 minutes. To stain for Group A Carbohydrate (GAC), unique to *Spy*, samples were incubated with goat anti-GAC antibody (Fitzgerald 70-XG70_R) for 1 hour, then rabbit anti-goat APC (Invitrogen A56570) for 1 hour. Samples were analyzed using BD FACSymphony A3 with excitation lasers: FITC, PE-594-A, APC, APC-Cy7. Appropriate single-color controls were used as compensation controls for these experiments. Data was analyzed using FlowJo, RRID:SCR_008520. For analysis, *speB* expressors were determined based on fluorescent intensity. Non-expressors were determined by background fluorescence from the *Spy* empty vector negative control, below 10^2^. Low expressors were identified between the ranges of 10^2^ and 10^3^, whereas high expressors were identified above 10^3^.

### Protein expression and purification:

Expression plasmid pETxSUMO-Vfr was introduced to *Escherichia coli* Rosetta (DE3) and induced at 37°C for 3 hour with 1 mM isopropyl-ß-D-thiogalactopyranoside (IPTG) when O.D. 600 reached 0.4–0.6. Cells were pelleted resuspended in buffer (20 mM Tris-HCl pH 8.0) and disrupted with sonication. Lysates were centrifuged at 20,000 × g 10 minutes at 4°C. Inclusion bodies were washed twice with wash buffer 1 (20 mM Tris-HCl, 2 M urea, 0.3 M NaCl, 2% TritonX-100 pH 8.0) and dissolved in binding buffer (20 mM Tris-HCl, 0.5 M NaCl, 5 mM imidazole, 6 M guanidine hydrochloride, 1 mM 2-mercaptoethanol pH 8.0). Sample was mixed at low speed for 40 minutes at room temperature and then filter-sterilized through 0.22 μm filter (Avantor). Filtered sample was loaded to a washed and equilibrated HisTrap HP column (Cytivia) with 0.5 ml NiSO_4_. Sample within the column was washed with binding buffer and wash buffer 2 (20 mM Tris-HCl, 0.5 M NaCl, 6 M urea, 1 mM 2-mercaptoethanol pH 8.0). Refolding was performed with FPLC (AKTA start) using a urea linear gradient up to 6 M urea. Gradient volume was 30 mL and flow rate was 1 mL/minute. Sample was eluted using an imidazole linear gradient up to 400 mM imidazole in elution buffer (20 mM Tris-HCl, 0.5 M NaCl, 400 mM imidazole, 1 mM 2-mercaptoethanol pH 8.0). Gradient volume was 10 mL and flow rate was 0.5 mL/minute. Fractions containing eluted protein were pooled and quantified. SpeB was purified as previously described ^38,43^.

### Vfr Cleavage Assay:

For *in vitro* cleavage experiments, recombinant Vfr (rVfr) (0.3 mg/mL) was incubated with titrations of purified SpeB (0.1 – 25 μg/mL) or recombinant Neutrophil Elastase (1 μg/mL; Sigma 324681) in assay buffer (Tris 25 mM, 300 mM NaCl, 2 mM dithiothreitol) at 37°C for 2 hours. Proteins and their cleavage products were then separated by SDS-PAGE and visualized by AcquaStain (Bulldog Bio). For neutrophil protein cleavage, purified rVfr (0.3 mg/mL) was incubated with neutrophil soluble protein (as described above) from 10^6^ cells/mL in RPMI supplemented with 2 mM dithiothreitol for 12 hours. Protease inhibitors AEBSF, Chymostatin, and PMSF were used as per manufacture protocol (G Biosciences 786–207). Neutrophil Elastase selective protease inhibitor BAY-687 (Cayman 18615) was used in titrating quantities (0.1 – 20 μg/mL).

### Protein Modeling and Cleavage Prediction:

The structure of Vfr was modeled as a dimer in AlphaFold Server v3 and visualized in PyMOL, RRID:SCR_000305. Sites of potential SpeB cleavage were predicted as previously described based on known targets^44^ using ScanProsite (Expasy) with a search for the motif [IVFYM]-[ADEGKSTN] and the included condition for a net negative charge sidechain charge in the P1’-P5’ region. Sites of NET cleavage are from the motif previously described^28^.

### Protein Fractionation:

Neutrophils isolated from whole blood (as above) were pelleted at 2,000 × g for 5 minutes and washed in Tris 20 mM, 1 M NaCl pH 8.0, then disrupted with sonication on ice. Neutrophil lysate was centrifuged at 20,000 × g at 4°C and filter sterilized through 0.22 μm filter (Avantor) then separated by anionic exchange by FPLC (Cytiva). Neutrophil protein was fractioned with a linear NaCl gradient up to 1 M NaCl in 20 mM of Tris. Fractions were assessed by SDS-PAGE and assayed with *Spy* containing GFP (*PspeB::gfp*) fluorescent reporter.

### Protease activity:

Internally-quenched FRET peptides IFFDTWDNE, TWDNEAYVH, EAYVHDAPV, and HDAPVRSLN that detect neutrophil proteases^45^ were pooled (2.5 μM each) in PBS, 1 mM CaCl*2*, 0.01% Tween-20 (Sigma P7949) and unincubated with each column fraction. After 1 hour incubation at 37°C, the proteolysis was measured using an Nivo plate reader (PerkinElmer) with fluorophore excitation at 323 nm and emission at 398 nm as previously^45^. Measurements of SpeB activity within bacterial supernatants was performed as previously with the fluorescent peptide sub103, internally quenched with an N-terminal Mca and the C-terminal Lys-Dnp (CPC Scientific)^43^. In triplicate, 10 μM of peptide was incubated in assay buffer (PBS with 2 mM dithiothreitol) with 10 μL of supernatant at 37°C for 30 minutes. Kinetic fluorescence was measured every 30 sec (ex. 323 nm, em. 398 nm) using a Victor Nivo plate reader (PerkinElmer).

### Statistics:

GraphPad Prism, RRID:SCR_002798, was used to evaluate statistical significance. Unless otherwise stated, one-way ANOVA with Dunnett’s multiple comparisons test was used for the statistical analysis of experiments and P values < 0.05 were considered significant.

## Supplementary Material

Supplement 1

## Figures and Tables

**Table 1:** Strain List

*S. pyogenes* strain	Description	Reference

M1 5448	Wild-type M1	Ref^35^
5448ΔcovR	Deletion of *covR*	This study
5448ΔcovS	Deletion of *covS*	This study
5448ΔropB	Deletion of *ropB*	This study
5448ΔspeB	Deletion of *speB*	Ref^36^
5448Δvfr	Deletion of *vfr*	This study

**Table 2:** Cloning Primers

Primer name	Application	Sequence

pDCerm_GFP A	GFP construct on pDC::erm backbone	5′-ctaatgttgtgaatgttaataaggtATGAGTAAAGGAGAAGAACTTTTCA
pDCerm_GFP B	GFP construct on pDC::erm backbone	5′-gaggaaacaaggaaatgaagCTATTTGTATAGTTCATCCATGC
pDCerm_RFP A	RFP construct on pDC::erm backbone	5′-ctaatgttgtgaatgttaataaggtATGGTTTCAAAAGGTGAAGAAGA
pDCerm_RFP B	RFP construct on pDC::erm backbone	5′-gaggaaacaaggaaatgaagCTATTTGTAAAGTTCATCCATACCA
pDCerm _GFP/RFP C	GFP/RFP construct on pDC::erm backbone	5′ - accttattaacattcacaacattag
pDCerm _GFP/RFP D	GFP/RFP construct on pDC::erm backbone	5′-cttcatttccttgtttcctcctag
PspeB-GFP A	PspeB controlled GFP on pDC::erm backbone	5′ - ctaatgttgtgaatgttaataacaagccttcctagttg
PspeB-GFP B	PspeB controlled GFP on pDC::erm backbone	5′ -gaaaagttcttctcctttactcattttttttatacctc
PspeB-GFP C	PspeB controlled GFP on pDC::erm backbone	5′ -ttattaacattcacaacattagcgg
PspeB-GFP D	PspeB controlled GFP on pDC::erm backbone	5′ -atgagtaaaggagaagaacttttc
PhasA-RFP A	PhasA controlled RFP on pDC::erm backbone	5′-ctaatgttgtgaatgttaataatcagatgaagttgt
PhasA-RFP B	PhasA controlled RFP on pDC::erm backbone	5′-ttcttcaccttttgaaaccataattacacctctttctt
PhasA-RFP C	PhasA controlled RFP on pDC::erm backbone	5′ -ttattaacattcacaacattagcgg
PhasA-RFP D	PhasA controlled RFP on pDC::erm backbone	5′ -atggtttcaaaaggtgaagaa
PspeB_PhasA A	Dual GFP and RFP Reporter Construct	5′ -ttcgcattacctgacggaGATATCTAGAGCTCCGCGG
PspeB_PhasA B	Dual GFP and RFP Reporter Construct	5′ -gcagtgacagagtacgctCGCTAGGAGGAAACAAGGA
PspeB_PhasA C	Dual GFP and RFP Reporter Construct	5′ -tccgtcaggtaatgcgaa
PspeB_PhasA D	Dual GFP and RFP Reporter Constructt	5′ -agcgtactctgtcactgc
ΔcovS F	*covS* deletion construct	5′ -TTGTACAAAAACGATAAAACACATCTGAGAATTGATGAC AGAAAGGGCAGTCGAGTCATTAGGAGTGAGCGCGAT GATCCTCGAGCTCTAGATCTTAAGC
ΔcovS R	*covS* deletion construct	5′ -AGCCTGGGTACTTGTTCGATTACGTGATTTATCCGTTCTAT AAAAGCGTTCAAAGATATGTTCCATGGCGCGCTTACCAAT TAG
ΔcovR F	*covR* deletion construct	5′ -ACTGCTTTGGAAAAAGAGTTTGATTTAATCCTGCTTG ACTTAATGTTACCAGAGATGGATGGTTTTGAAGGATC CTCGAGCTCTAGATCTTAAGC
ΔcovR R	*covR* deletion construct	5′ -CACTGTTTGGATATAAGATTCCTTGCCTGGAATGTCA ATTTTGCCGCGGAGATAACGAATATAGACATCTCATG GCGCGCTTACCAATTAG
ΔropB F	*ropB* deletion construct	5′ -AACCGTTGAATTCATTAGGCATTCAAAAAACATTTCGATT AAACAAGTTTGTGGTGATTATCTCACTAGGCAAACGATCC TCGAGCTCTAGATCTTAAGC
ΔropB R	*ropB* deletion construct	5′ -TCAGGACAGTTTATGTTTAATGGCTTCTAGGTAGGTTTGA AACATATGATGGATCGTTTTGCAATTAAGTAATTGCATGG CGCGCTTACCAATTAG
Δvfr F	*vfr* deletion construct	5′ -TCATAGTTAGGTTTACCGATTTTACTTGACGCCTTAAT GATGACATGCTAGTATTTTATATTAAGGAGATGAGTC GATCCTCGAGCTCTAGATCTTAAGC
Δvfr R	*vfr* deletion construct	5′ -AAACTTCTTCAATTGAATAATTTTCAAAACATGAAAA AATCCGTCTTGTGACTACGTTATACACTTGACGGACTT CATGGCGCGCTTACCAATTAG
pET-SUMO-vfr-F	vfr expression plasmid	CTCACAGAGAACAGATTGGTGGTATGACACAAGTAGCCC AAGG
pET-SUMO-vfr-R	vfr expression plasmid	TGCTCGAGTGCGGCCTCA GAGTAATCCTTTTGAAAAAGAAGTATGTC

**Table 3:** Plasmids

Plasmid name	Description	Reference

pDCerm	Reporter backbone	Ref^37^
pGFP	Plasmid with *gfp* template	GenBank: OM212390.1
pRFP	Plasmid with *rfp* template	GenBank: KM521211.1
pDCerm GFP	Reporter construct	This study
pDCerm mCherry	Reporter construct	This study
pDCerm-PspeB::GFP	*speB* expression reporter	This study
pDCerm-PhasA-mCherry	*hasA* expression reporter	This study
pDCerm-PspeB::GFPPhasA::mCherry	Dual *speB* and *hasA* expression reporter	This study
pET-SUMO	Expression vector	Ref^38^
pET-SUMO Vfr	Vfr expression vector	This study
pAV488	Recombineering plasmid	Ref^39^
