# Supplementary material for: A protease-sensing circuit links neutrophil inflammation to virulence regulation in Streptococcus pyogenes
Source: bioRxiv. 2026 May 15:2026.05.15.725401. Preprint. [Version 1] doi: 10.64898/2026.05.15.725401 (PMC13193001; doi:10.64898/2026.05.15.725401)
Supplement: Supplement 1 [file NIHPP2026.05.15.725401v1-supplement-1.pdf]

bioRxiv preprint doi: https://doi.org/10.64898/2026.05.15.725401; this version posted May 15, 2026. The copyright holder for this preprint (which was not certified by peer review) is the author/funder, who has granted bioRxiv a license to display the preprint in perpetuity. It is made available under a CC-BY 4.0 International license.

Supporting Information Legends

Fig. S1. (A) Wild-type *Spy* growth kinetics determined through optical density at 600 nm (O.D. 600) in RPMI, 5% THY with LL-37 (300 nM) or $MgCl_2$ (15 mM). (B) SpeB activity was measured using the fluorescent peptide sub103. (C) Flow cytometry gating strategy for measuring *Spy* GFP and RFP fluorescence. Samples were selected based on particle size (FSC-Area, SSC-Area), then selected for into single cells (FSC-Area, Height; SSC-Area, Height). Sample containing Group A Carbohydrate (APC-A positive population; right peak) were selected. Lastly, live cell population was selected (APC-Cy7-A negative population; left peak). (D) Flow cytometry demonstrating *speB* expression (GFP; horizontal axis) and *hasABC* expression (RFP; vertical axis) of *Spy* growing at stationary phase separated in panels based on treatment.

Fig. S2. (A) *Spy* growth kinetics determined through optical density at 600 nm (O.D. 600) in RPMI, 5% THY. (B) Lionheart live-cell fluorescent microscopy with brightfield, GFP, and RFP channels on of *Spy* culture grown at stationary phase. Scale bars, 100 µm. (C) Measurement of fluorescence over cell density after 10 h of growth. Wild-type and Δ*vfr Spy* were treated with LL-37 (300 nM) or $MgCl_2$ (15 mM).

Fig. S3. (A) Regulation of *speB* and *hasABC* during mouse intradermal and human blood infections. Flow cytometry demonstrating *speB* (GFP fluorescence; horizontal axis) and *hasABC* induction (RFP fluorescence; vertical axis) of 10^8 CFU of *Spy strains.* (B) Colony Forming Units (CFU) of *Spy* within 4 h human blood infection was measured by plating. (C) SDS-PAGE of Vfr (0.3 mg/mL) incubated with recombinant Neutrophil Elastase (rNE) or neutrophil lysate (10^6 cells/mL) with inhibitor BAY-678. (D) Measurement of RFP fluorescence (*hasABC*) over cell density after 10 h of growth. (E) *Spy* growth kinetics determined through optical density at 600 nm (O.D. 600).

bioRxiv preprint doi: https://doi.org/10.64898/2026.05.15.725401; this version posted May 15, 2026. The copyright holder for this preprint (which was not certified by peer review) is the author/funder, who has granted bioRxiv a license to display the preprint in perpetuity. It is made available under a CC-BY 4.0 International license.

Fig. S4. (A) Flow cytometry gating strategy for neutrophil depletion model with anti-Ly6G. Singlets were selected through side (SSC-H, SSC-W) and forward (FSC-H, FSC-W) scatter. Population of live cells were selected based on BUV480-A fluorescence (left). Granulocytes were selected based on the presence of CD45, and neutrophils were identified based on presence of Ly6G and CD11. (B, C) Flow cytometry demonstrating *speB* (GFP fluorescence; horizontal axis) and *hasABC* induction (RFP fluorescence; vertical axis) of 10^8 CFU of *Spy*. *Spy*Δ*vfr* strain in anti-Ly6G neutrophil depletion model (B). Control strains, *Spy* empty vector and Δ*vfr*, during mouse intradermal infection with $PAD4^{-/-}$(C).
